# *DEP* and *AFO* Regulate Reproductive Habit in Rice

**DOI:** 10.1371/journal.pgen.1000818

**Published:** 2010-01-22

**Authors:** Kejian Wang, Ding Tang, Lilan Hong, Wenying Xu, Jian Huang, Ming Li, Minghong Gu, Yongbiao Xue, Zhukuan Cheng

**Affiliations:** 1 State Key Laboratory of Plant Genomics, Institute of Genetics and Developmental Biology, Chinese Academy of Sciences, Beijing, China; 2 Laboratory of Molecular and Developmental Biology, Institute of Genetics and Developmental Biology, Chinese Academy of Sciences, Beijing, China; 3 Key Laboratory of Plant Functional Genomics of Ministry of Education, Yangzhou University, Yangzhou, China; Iowa State University, United States of America

## Abstract

Sexual reproduction is essential for the life cycle of most angiosperms. However, pseudovivipary is an important reproductive strategy in some grasses. In this mode of reproduction, asexual propagules are produced in place of sexual reproductive structures. However, the molecular mechanism of pseudovivipary still remains a mystery. In this work, we found three naturally occurring mutants in rice, namely, *phoenix* (*pho*), *degenerative palea* (*dep*), and *abnormal floral organs* (*afo*). Genetic analysis of them indicated that the stable pseudovivipary mutant *pho* was a double mutant containing both a Mendelian mutation in *DEP* and a non-Mendelian mutation in *AFO*. Further map-based cloning and microarray analysis revealed that *dep* mutant was caused by a genetic alteration in *OsMADS15* while *afo* was caused by an epigenetic mutation in *OsMADS1*. Thus, *OsMADS1* and *OsMADS15* are both required to ensure sexual reproduction in rice and mutations of them lead to the switch of reproductive habit from sexual to asexual in rice. For the first time, our results reveal two regulators for sexual and asexual reproduction modes in flowering plants. In addition, our findings also make it possible to manipulate the reproductive strategy of plants, at least in rice.

## Author Summary

Sexual reproduction is essential for the life cycle of most flowering plants. However, pseudovivipary, in which floral organs are replaced by bulbils or plantlets, provides an asexual means for many grasses to reproduce in extreme environments. Although the molecular mechanism of pseudovivipary is still unknown, the high-frequency occurrence of pseudovivipary in extreme environments indicates that only a few key regulators are responsible for the switch of reproductive habit. Here, by analyzing three naturally occurring mutants in rice, we show that mutations in *DEP* and *AFO* lead to the transformation of rice flowers/spikelets into juvenile plantlets and subsequently the switch of reproductive strategy from sexual to asexual, suggesting that *DEP* and *AFO* might work cooperatively to regulate reproductive habit in rice. Thus, we reveal a critical mechanism of the switch of reproductive habit in plants. In addition, our results also make it possible to manipulate the reproductive habit of plants, at least in rice.

## Introduction

Flowering is an important process essential for sexual reproduction, seed development and fruit production. Although flowering is composed of a series of typically irreversible sequential events, reversion from floral to vegetative growth is frequently observed in nature. Reversions can be divided into two categories: inflorescence reversion, in which vegetative growth is resumed after or intercalated within inflorescence development, and flower reversion, in which vegetative growth is resumed in an individual flower [1],[2]. Reversion, which can serve a function in the life history strategy (perenniality) or reproductive habit (pseudovivipary), is essential for the life cycle of some plant species [1],[2].

Vivipary in flowering plants is defined as the precocious and continuous growth of the offspring while still attached to the parent plant [3],[4]. Vivipary can be divided into two distinct types: true vivipary and pseudovivipary [3]. True vivipary is a sexual reproduction process in which seeds germinate before they detach from maternal plant. On the other hand, pseudovivipary is a specific asexual reproductive strategy in which bulbils or plantlets replace sexual reproductive structures [3],[5]. Pseudovivipary has been widely recorded in monocots, in particular grasses that grow in extreme environments [1], [3], [5]–[11]. Characteristics of the environments which favour pseudovivipary include climate changes, high precipitation and humidity, drought, fungal infection, high altitudes and latitudes, late-thawing habitats, or arid/semi-arid areas [1],[3],[5]. Several authors have argued that pseudovivipary has evolved in response to a short growing season, enabling plants to rapidly complete the cycle of offspring production, germination and establishment during the brief periods favourable to growth and reproduction [3]. In developmental terms pseudovivipary occurs in two principal ways. The first way to proliferate, as in *Festuca ovina*, *Poa alpina* and *Poa bulbosa*, is through the transformation of the spikelet axis into the leafy shoot. The second way is to form the first leaf of the plantlet by lemma elongation, as is the case in *Deschampsia caespitose* and *Poa robusta* [1],[11]. In some cases, such as *Deschampsia alpine* and *Phleum pratense*, both modes of propagule development have been found in a single plant [11], indicating that the molecular difference between the two types of pseudovivipary might be rather small.

Pseudovivipary has fascinated biologists, as elucidation of its mechanism could lead to an understanding of flower evolution and sexual reproduction; hence, it might provide an opportunity to manipulate a plant's reproductive strategy. As pseudovivipary is always closely associated with various environmental factors, the molecular basis of pseudovivipary is still unknown. Here we report mutations of two MADS-box transcription factors that are essential for sexual reproduction and mutations of which lead to stable pseudovivipary in rice.

## Results

### Characterization of *pho* mutant

**Figure 1:**
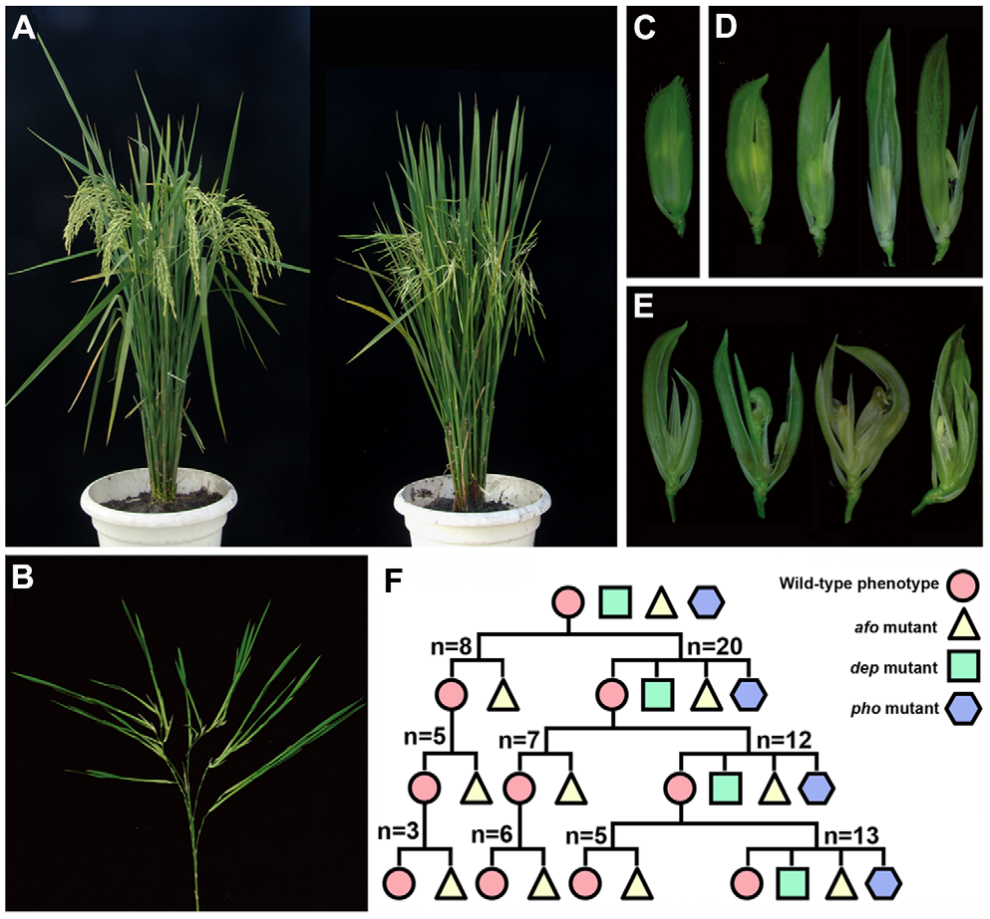
Phenotypic characterization and genetic analysis of *pho*, *dep*, and *afo* mutants. (A) The phenotype of wild-type (left) and *pho* (right) plants. (B) All flowers are replaced by young plantlets in *pho* panicle. (C) The spikelet of wild-type rice. (D) The spikelets of *dep* in the order of increasing severity showing the defects of paleas. (E) The spikelets of the *afo* mutant showing pleiotropic defects in lemmas and the inner three whorls. (F) Genetic analysis of *pho*, *dep*, and *pho* mutants indicates that *pho* might be a double mutant containing both a Mendelian mutation in *DEP* and a non-Mendelian mutation in *AFO*; “n” indicates the line number.

In this study, a naturally occurring mutant showing inflorescence reversion was found in the offspring of an *Oryza sativa* spp. *indica* var. Zhongxian 3037. Instead of normal floral organs, this mutant generated new plantlets (Figure 1A and 1B). The plantlets, like normal juvenile plants, generated roots, produced tillers and showed normal vegetative growth when explanted in paddy fields (Figure S1A and S1B). In the subsequent life cycle, plants again displayed inflorescence reversion. Thus, this mutant could be considered to be a complete pseudovivipary mutant in which the reproductive mode has completely changed from sexual to asexual. In fact, this mutant has accomplished six life cycles via this asexual reproductive method. This type of mutation has not been reported before in rice. We named the mutant *phoenix* (*pho*) to reflect its stable “never die and reborn anew” phenotype. Two additional mutants were also found in this segregating population. The first mutant was named *degenerative palea* (*dep*), and was characterized by shrunken paleas. Paleas in severe *dep* florets degenerated to glume-like organs that were prone to splitting. The lemmas and glumes in *dep* florets were slightly elongated (Figure 1D). The second mutant *abnormal floral organs* (*afo*) displayed a phenotype opposite to *dep*, with abnormalities primarily in lemma and the inner three whorls (Figure 1E).

In order to examine the genetic basis of the three mutations, seeds of the 28 individual plants showing the normal phenotype from the above population were planted into lines by parent plants. We found that those genotypes self-segregated into two categories. The first category only produced *afo* and wild phenotype plants, while the second category produced *dep*, *afo*, and *pho*, as well as wild phenotype plants. As the segregation ratios in both categories seemed unclear, seeds of the wild phenotype plants from each category were planted in individual lines for two more generations. Subsequently, all plants in the final generation were counted and analyzed (summarized in Figure 1F). In the first category lines, 35.34% of plants displayed the *afo* phenotype, while 64.66% of plants exhibited the wild phenotype (n = 232). As the segregation did not follow Mendelian patterns (3∶1 ratio, *χ* ^2^ (1) = 13.24, *P*<0. 01), we proposed that *afo* might be a non-Mendelian mutant. In the second category lines, 28.44% plants showed the *afo* phenotype, 18.35% plants showed the *dep* phenotype and 7.34% plants showed the *pho* phenotype (n = 218). We observed that *pho* only appeared in the line where *afo* and *dep* mutants coexisted. In addition, when we put the wild phenotype plants and *afo* mutants into one group and *dep* and *pho* into another group, the segregation ratio would fit a 3∶1 ratio (162∶56, *χ* ^2^ (1) = 0.06, *P*>0.50), indicating that *dep* might be a Mendelian mutant. Therefore, we further hypothesized that *pho* might be a double mutant containing both a Mendelian mutation in *DEP* and a non-Mendelian mutation in *AFO*.

### Single amino acid mutation disrupts the transcriptional activation of OsMADS15 in *dep*

**Figure 2:**
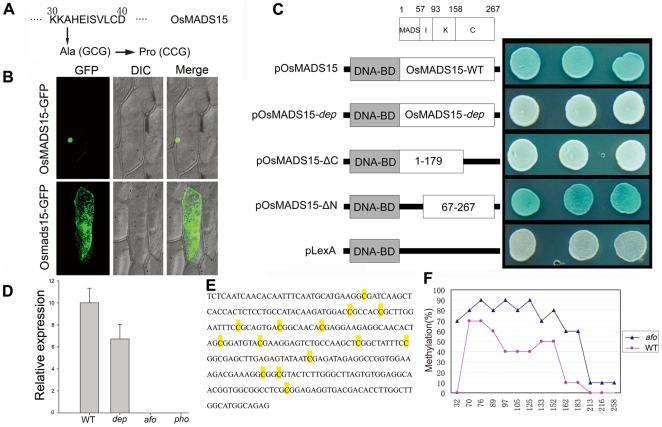
Molecular mechanisms of *dep* mutant and *afo* mutant. (A) Amino acid mutation corresponding to the nucleotide change in *dep*. (B) OsMADS15-GFP fusion protein is localized in nucleus while Osmads15 (*dep*)-GFP fusion protein is localized in cytosol. (C) Transcriptional activation assay of pOsMADS15, pOsMADS15-*dep*, pOsMADS15△C180-267, pOsMADS15△N1-66, and pLexA. White clones indicate no activation of the reporter gene while blue clones indicate activation of the reporter gene. (D) *OsMADS1* expression analysis by quantitative real-time PCR analysis in WT, *dep*, *afo*, and *pho* panicles shows the silencing of *OsMADS1* in *afo* and *pho*. (E) 294-bp sequence in the promoter region of *OsMADS1* gene shows different cytosine methylation in WT and *afo*. The yellow-marked cytosines were found to be methylated in WT or *afo*. (F) Profiles of DNA methylation in 294-bp region in WT (red line) and *afo* (blue line) plants. The numbers on the X axis represent cytosine positions in the analyzed region, and the Y axis represents methylation ratios in WT and *afo*.

To understand the molecular mechanism of pseudovivipary in *pho*, we began by isolating the *DEP* gene through map-based cloning. The *dep* mutants from the second category line were crossed to *O. sativa* spp *japonica* var. Zhonghua11 to generate a mapping population. In the F2 population, 71 of 302 plants showed the *dep* phenotype (3∶1 ratio, *χ* ^2^ (1) = 0.36, *P*>0.50), confirming that the phenotype of the *dep* mutant is controlled by a single recessive gene. 2,292 F2 and F3 plants showing the *dep* phenotype were used to map *DEP* to a 50-kbp region on the short arm of chromosome 7. All genes within this region were amplified and sequenced. A single nucleotide G to C substitution at position 94 in coding region was found in the first exon of the *OsMADS15* in the *dep* mutant. This substitution results in a change from a MADS-box conserved alanine residue to proline (Figure 2A and Figure S5). The same nucleotide mutation was also found in all the *pho* mutants analyzed (n = 20), further implying that the mutation of *OsMADS15* might be partly responsible for the *pho* phenotype. To confirm that the loss of function of OsMADS15 is responsible for *dep*, we utilized an RNA interference approach to down-regulate *OsMADS15*. Forty transgenic plants expressing an inverted repeat of 317 bases of *OsMADS15* were generated in Nipponbare. Among them, 35 plants also displayed the *dep* degenerative palea phenotype (Figure S1C and S1D). Therefore, we concluded that the phenotype of the *dep* mutant is indeed caused by mutation in *OsMADS15*.

We found five *OsMADS15* transcripts with differing sequences in GeneBank. To identify the WT *DEP* sequence, we performed RT-PCR and found that our cDNA sequence was identical to GB accession AB003325. This cDNA was used for subsequent analysis. MADS-box proteins are transcription factors, so we conducted experiments to evaluate whether amino acid substitution impaired the transcriptional activation function of OsMADS15 in the *dep* mutant. OsMADS15 from both WT and *dep* were fused with GFP protein and transiently expressed in onion epidermal cells as well as rice protoplast cells. The OsMADS15 GFP signal was localized in the nucleus, whereas the *dep* mutant caused redistribution of OsMADS15 GFP to the cytosol (Figure 2B and Figure S2). Previous study has revealed that the KC region of OsMADS15 (Amino acids of AF058698) does not show any transcriptional activation function [12]. However, a single amino acid substitution, from leucine to histidine mutation, has occurred at position 117 of the amino acids of AF058698. In our study, we found that the OsMADS15 protein itself exhibited transcriptional activator activity. Furthermore, when the MADS domain of OsMADS15 was eliminated, the residual IKC region of OsMADS15 also displayed transcriptional activator activity. However, the mutated protein in *dep* lost its transcriptional activator activity completely, though the amino acid mutation only occurred in the MADS domain (Figure 2C). Taken together, it is very likely that the mutated OsMADS15 protein has lost its transcriptional activation function in *dep*.

### *afo* is an epigenetic mutant of *OsMADS1*, while *pho* is a spontaneous mutant containing both genetic mutation in *OsMADS15* and epigenetic mutation in *OsMADS1*

From the above genetic analysis, it was deduced that *pho* and *afo* were non-Mendelian mutants, so we proposed that they might be epigenetic mutants. Epigenetic mutations are often marked by a reduction or elimination of an associated transcript. Microarray experiments were carried out to investigate whether there were any variations in transcript accumulation between *pho* and WT young panicles (Table 1). These experiments showed that the transcript levels of multiple genes were altered. Of those altered genes, *OsMADS1* (also known as *LEAFY HULL STERILE1*, *LHS1* [13]), was the most significantly altered transcript, with a 2,208-fold reduced expression in *pho* relative to WT. Real-time PCR was further performed using WT, *dep*, *afo* and *pho* panicle transcripts to confirm this result and to examine whether the *afo* mutant also showed a reduced expression of *OsMADS1* transcripts. As expected, the expression of *OsMADS1* was hardly detectable in *afo* as well as *pho* (Figure 2D). Additionally, no mutations were detected in the 12,879-bp genomic sequence of the *OsMADS1* locus, including the eight exons, seven introns, 2,507-bp upstream sequence and 1,870-bp downstream sequence. We hypothesized that the *afo* mutant might be caused by an epigenetic modification of *OsMADS1*. Interestingly, recent studies in hexaploid wheat (*Triticum aestivum*) revealed that *WLHS1-B*, one of the homologs of *OsMADS1*, was silenced by cytosine methylation [14]. To test if this was also the case in rice, we used bisulfate sequencing of exon 1 and the 5′ upstream regions of *OsMADS1* in *afo* to characterize their methylation status. Compared with the WT plants, the promoter region of *OsMADS1* in *afo* was more heavily methylated (from 31.43% to 62.86%), which might contribute to the silencing of *OsMADS1* (Figure 2E and 2F).

**Table 1:** Expression analysis of MADS-box genes in *pho* mutant according to the microarray data.

Gene	WT Signal	*pho* Signal	WT-vs-*pho* Signal Ratio
*OsMADS1*	10165.8	4.6	2209.96
*OsMADS8/24*	9202.9	156.7	58.73
*OsMADS7/45*	8547.8	194.6	43.92
*OsMADS29*	119.6	4.1	29.17
*OsMADS13*	247.9	14	17.71
*OsMADS17*	3034.6	318.6	9.52
*OsMADS4*	1842.2	224.9	8.19
*OsMADS3*	414.3	103.2	4.01
*OsMADS5*	7152.3	1577.8	4.53
*OsMADS6*	5314.5	1423.5	3.73
*OsMADS2*	6275	2591.8	2.42
*OsMADS14*	713	2063.4	0.35
*OsMADS34*	569.7	2269.7	0.25

To ascertain whether *pho* was a *dep/afo* double mutant, We crossed *dep* with *naked seed rice* (*nsr*), a mutant of the *OsMADS1* gene [15], to generate *dep/nsr* double mutants. In the F_2_ and F_3_ population, all the *dep/nsr* double mutants analyzed (n = 35) showed a similar pseudovivipary phenotype to that of the *pho* mutants (Figure S3). This double mutant has accomplished three life cycles via asexual reproductive method. So, this result confirmed that *pho* was a double mutant of *Osmads1* and *Osmads15*.

### *dep* displays pseudovivipary occasionally

The spikelet development of each of the three mutants was further analyzed to explore functions of the two MADS-box genes during spikelet development. Previous studies have characterized *OsMADS1* as a *SEPALLATA* (*SEP*)-like gene and performed multiple investigations in rice. However, the function of *OsMADS1* is still not fully elucidated [13], [15]–[20]. The *afo* mutant shared many similarities with those severely affected *Osmads1* (*lhs1*) mutants and *OsMADS1*RNAi plants (Figure 1E): all spikelets were sterile; lemmas were more severely affected than paleas; palea marginal tissues (PMTs) were absent while palea main structures (PMSs) were only slightly effected; lodicules were converted into glume-like organs; and ectopic florets that are indicative of partial reversion had frequently arisen from the parent florets [13],[15],[19],[20]. In summary, the phenotype of *afo* mutant suggests that *OsMADS1* is required for the specification of lemma, PMTs and the three inner whorls [13],[15],[19],[20]. Its pleiotropic defects indicate that *OsMADS1* is essential for flower meristem (FM) determinacy [13], [15], [19]–[22].

**Figure 3:**
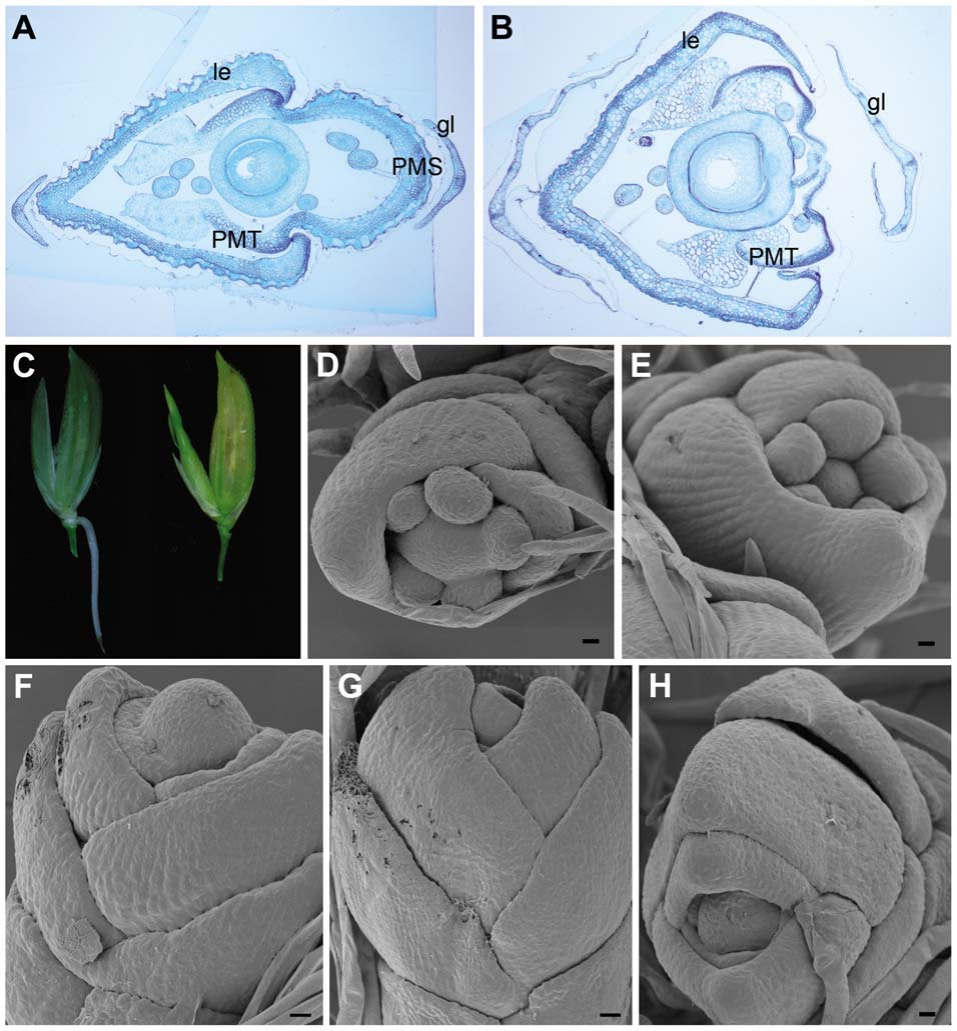
Spikelet morphologies of WT, *dep*, *afo*, and *pho* plants. (A) Transverse section of the WT spikelet shows normal glumes (gl), lemma (le), palea main structure (PMS) and palea marginal tissue (PMT). (B) Transverse section of the severely affected *dep* spikelet shows the loss of PMS. (C) Occasional emergence of root at the base of *dep* rachilla on the lemma side (left) and occasional emergence of tiller between palea and upper empty glume in *dep* spikelets (right, see also Figure S7). (D,E) SEM of the floral primordium in WT shows that only two empty glumes, lemma (le) and palea (pa) are arranged in alternate phyllotaxis. (F–H) SEM of the floral primordium in *pho* shows that all lateral organs are arranged in alternate phyllotaxy. Bars in (A,B), 200 µm; bars in (D–H), 10 µm.

Phylogenetic analyses have characterized *OsMADS15* as an *APETALA1* (*AP1*)/*FRUITFUL* (*FUL*)-like gene (Figure S4 and Figure S5) [21]–[23]. In addition, previous study has shown that *OsMADS15* (*RAP1A*) RNA was expressed in the incipient floral primordium and later mainly accumulated in empty glumes, lemma, palea and lodicules [23]. However, the function of *OsMADS15* is still unclear [21],[22]. The effects of *OsMADS15* on cell specifications of all spikelet whorls were histologically examined. In a severely affected *dep* spikelet, the transformed palea was actually only composed of two PMTs while the PMS was completely lost (Figure 3A and 3B). This implied that the identity of palea was lost in the *dep* spikelet with the severe phenotype. The lemma in the *dep* spikelet was also slightly affected, but its identity was still maintained (Figure 3A and 3B, and Figure S6). The glumes of *dep* spikelets contained many more bundles than the WT glumes, suggesting a possible partial reversion of glumes to leaf-like organs. No obvious difference was found in the inner three whorls, hinting that they are not affected by the mutation of *OsMADS15*. Thus, *OsMADS15* is required for the specification of PMS and empty glumes, those floral organs are just opposite to the affecting whorls of *OsMADS1*.

*dep* showed a stable degenerative palea phenotype when grown in paddy fields with a normal climate. Unexpectedly, however, we found that, under a continuous rain for several days during its heading stage, roots occasionally emerged from the base of *dep* rachillas (Figure 3C). Only one root was formed in each spikelet and it merely located at the lemma side (n = 22). These roots would soon degenerate if the spikelets were dried. Interestingly, if the continuous rain occurred after the heading stage, the inner floral organs or developing seeds of *dep* always got mildewed because of the lack of protection by paleas, but emergence of new shoots was occasionally visible in *dep* spikelets (Figure 3C and Figure S7A, S7B, and S7C). In contrast to the emerged roots that were only formed on the lemma side, these emerged shoots only appeared between paleas and upper empty glumes on the other side (n = 24). Moreover, prophylls were found on these shoots, indicating that these emerged shoots are actually tillers. These tillers also generated roots, produced new tillers and showed normal vegetative growth when replanted in fields (Figure S7D and S7E). So, *dep* can also be considered to be an unstable pseudovivipary mutant that was closely associated with environmental factors. In the *dep* mutant, most floral organs develop normally, demonstrating that *OsMADS15* might only play a minor role in the FM determinacy. However, the occasional emergence of roots and tillers in *dep* implies that the shoot apical meristem (SAM) identity is restored and begins to grow under a suitable environment (continuous rain), so *OsMADS15* might also participate in inhibiting SAM formation in incipient floral primordium. However, pseudovivipary has not been observed in *DEP* RNAi plants that grow in paddy fields; it is probably that the residual transcripts in RNAi plants are sufficient to inhibit SAM formation in incipient floral primordium. Alternatively, pseudovivipary, which is mainly observed in natural plants, might be a *dep* allele–specific phenomenon.

Finally, the primordium development of *pho* mutant was also analyzed. In WT, two empty glumes, lemma and palea were arranged in alternate phyllotaxis while stamens and carpel were not (Figure 3D and 3E). In contrast, in the *pho* mutant, no stamen or carpel was observed and all lateral organs were arranged in alternate phyllotaxis (Figure 3F–3H). As those lateral organs finally grew into true leaves but not simple leaf-like organs, it is obvious that FM at least partially transformed into functional SAM although some following floral genes still expressed at this stage (Table 1).

## Discussion

### Pseudovivipary of *dep* and *pho* occurs in two distinct ways

Morphological studies in other grasses have revealed that pseudovivipary occurs either by proliferation of the spikelet axis or by transformation of the lemma [1],[11]. In most cases, pseudovivipary is achieved by the transformation of the spikelet axis.

**Figure 4:**
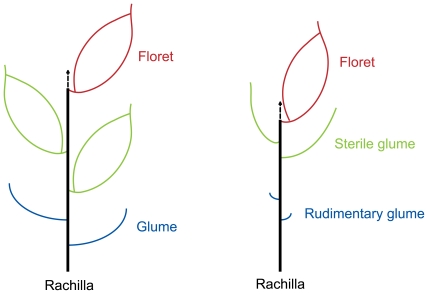
Diagrammatic representation of the spikelets of typical grass with three florets (left) and rice (right). The arrows indicate the spikelet axes, which are transformed to shoots in *dep* plants.

The grass spikelet is a structure consisting of two glumes subtending one or more small florets. The rice spikelet is generally considered to have three florets, which are subtended by two tiny glumes (rudimentary glumes) [21],[24]. The uppermost floret is fertile while the two lower florets are reduced and sterile. The two empty glumes (or sterile lemmas) are considered to be reduced lemmas of two lower florets [21],[24]. So, theoretically, rice spikelet axis is located between the palea and upper empty glume (Figure 4). In this study, new shoots in the *dep* mutant are merely found between paleas and upper empty glumes. Thus, we conclude that pseudovivipary in the *dep* mutant is also achieved by the transformation of the spikelet axis.

*Poa alopecurus* and *Poa fuegiana*, which are non-pseudoviviparous and pseudoviviparous species, respectively, can also be recognized as the same species because of the close affinities between them [11]. The characters of *Poa fuegiana* have been well described [11]. A detailed comparison of rice *dep* plant with *Poa fuegiana* shows that there are many similarities between the two pseudoviviparous plants: the palea is reduced or rudimentary; the lemma is elongated; new shoots are only formed on the palea side; both are not stable pseudoviviparous plants; and pseudovivipary mainly happens under high rainfall conditions. Considering so many similarities, it is very likely that the occurrence of pseudovivipary in *Poa fuegiana* and rice *dep* mutant might share the same mechanism. However, the validity of this speculation remains to be verified by molecular investigations on *Poa fuegiana*.

The *pho* mutant should be classified into the second type of pseudoviviparous plant since the lemma in *pho* undergoes elongation to form the first leaf of the propagule. However, *pho*, which differs from those environment-dependent pseudoviviparous grasses, shows stable pseudovivipary phenotype and is not associated with environmental factors. Till now, to our knowledge, no similar stable pseudoviviparous plant has been reported in nature. If similar stable pseudoviviparous plants are found in nature, they are very likely to be recognized as new species, because of the extreme difference in morphology and reproductive method.

### Roles of *OsMADS1* and *OsMADS15*

Early studies have showed that both *OsMADS1* and *OsMADS15* are expressed in the incipient floral primordium [16]–[18],[23]. Furthermore, *OsMADS1* interacts with *OsMADS15* in yeast two-hybrid experiments [12]. The defects of their mutants indicate that *OsMADS1* might work cooperatively with *OsMADS15* to determine FM, but their individual roles are divergent: *OsMADS1* mainly works in promoting the determinacy of FM while *OsMADS15* mainly functions in inhibiting the formation of SAM in incipient floral primordium. Consistent with those indications, the mutations of both *OsMADS1* and *OsMADS15* in *pho* result in a stable inflorescence reversion. In addition, *OsMADS1* is required for the specification of lemma, PMTs and three inner whorls. On the contrary, *OsMADS15* is required for the specification of PMS and empty glumes. So, it is also probably that all floral organs in the double mutant, *pho*, lost their modifications and transformed into their basal state, namely, leaves.

It has been shown that both transcripts of *OsMADS1* and *OsMADS15* are eventually accumulated in lemma and palea, suggesting that *OsMADS1* and *OsMADS15* might also be involved in the development of lemma and palea [17],[23]. In severely affected *Osmads1* spikelets, both lemma and palea are affected, but the lemma is affected to a greater extent, suggesting that *OsMADS1* might function as a lemma identity gene [19],[22]. Additionally, PMTs are lost in *Osmads1* spikelets, indicating that *OsMADS1* is also essential for the specification of PMTs. In contrast, in severely affected *Osmads15* spikelets, both lemma and palea are affected, but the palea is affected to a greater extent and PMS is completely lost, implying that *OsMADS15* might be mainly involved in the specification of PMS. Collectively, both *OsMADS1* and *OsMADS15* might control the differentiation of lemma and palea, but their different roles might contribute to the asymmetric development of the first whorl of rice spikelets.

*OsMADS1* and *OsMADS15* are characterized as *SEP*-like gene and *AP1*/*FUL*-like gene, respectively [12], [13], [15]–[23]. *AP1*, *FUL* and *SEP1/2/3/4* genes in dicot model plant *Arabidopsis* are also involved in floral meristem identity determination [25]–[28]. In addition, previous studies in *Arabidopsis* have transformed floral organs into leaf-like organs [26],[29],[30]. However, transformation of flowers into true plantlets that is indicative of pseudovivipary has not been found in *Arabidopsis*, but has been reported in many grasses in nature [1]. The difference might be caused by the distinction of floral development between grasses and dicot plants, as well as the diversification of those floral genes during evolution [16],[21],[31].

### Is grass flower a modified plantlet meant for reproduction?

More than 200 years ago, Goethe proposed that the floral organs are modified leaves. This belief is supported by the observation that triple mutants lacking the ABC genes in *Arabidopsis* have a conversion of all floral organs into leaf-like organs [29],[30]. In this study, we revealed that mutations in *OsMADS1* and *OsMADS15* lead to the transformation of all rice flowers into plantlets that can produce true leaves, thereby further confirming Goethe's hypothesis. The complete transformation of flowers into juvenile plantlets in rice, as well as similar transformations in other grasses, leads us to hypothesize that in grasses a flower may be a modified juvenile plantlet meant for reproduction.

It is widely accepted that sexual reproduction evolves from asexual reproduction, so we speculate that *pho* might be an atavistic mutant, and plants with similar phenotype might play an important role in the evolution of reproductive strategy from asexual to sexual. The *dep* mutant, which can produce both flowers and plantlets, is more similar to most natural pseudoviviparous plants than the *pho* mutant. Thus, its analogous plants might play an intermediate role in this evolution, because such environment-dependent pseudoviviparous plant has the ability not only to reproduce via sexual way under favourable conditions, but also to reproduce via asexual way when the harsh conditions affect its sexual reproduction.

In conclusion, we have shown that *dep* is a genetic mutant in *OsMADS15* while *afo* is an epigenetic mutant in *OsMADS1*, and their combination led to stable pseudovivipary. These findings suggest that the two MADS-box genes might play important roles in plant adaptation to various reproductive strategies.

## Materials and Methods

### Plant materials

All plant materials were grown in individual lines in paddy fields to monitor climate-change triggered pseudovivipary. In summer, all materials were planted in Beijing and Yangzhou, while, in winter, all materials were grown in Hainan Island in South China.

### Primers

The primers used in this study are listed in Table S1.

### Molecular cloning of *DEP*

To fine map *DEP*, STS markers (P1–P8) were developed based on sequence differences between *indica* variety 9311 and *japonica* variety Nipponbare according to the data published in http://www.ncbi.nlm.nih.gov.

### Construction of RNA interference and rice transformation

A 317-bp fragment of *OsMADS15* was amplified by PCR with their specific primers; this fragment was cloned into the pGEM-T vector (Promega) and sequentially cloned into the *Bam*HI*/Sal*I and *Bgl*II*/Xho*I sites of the pUCRNAi vector. Subsequently, the stem-loop fragment was cloned into the pCAMBIA2300-Actin vector. The resulting RNAi construct was transformed into an *A. tumefaciens* strain and used for further rice transformation.

### Subcellular localization

The amplified coding region of *OsMADS15* of both wild-type and *dep* was fused with green fluorescent protein (GFP) and cloned into the *Hind*III/*Bam*HI sites of the vector pJIT163. Those plasmids were bombarded into onion epidermal cells using a PDS-1000/He particle gun (Bio-Rad). The expression constructs were also transfected into rice Nipponbare protoplasts. Twenty hours after transfection, protein expression was observed and images were captured with a Zeiss LSM 510 Meta confocal laser scanning microscope.

### Transcriptional activation assay

We carried out the transcriptional activation assay using a MATCHMAKER LexA Two-Hybrid system (Clontech). Different length sequences were amplified and fused in frame to the pLexA to construct pOsMADS15, pOsMADS15-*dep*, pOsMADS15△C180-267 and pOsMADS15△N1-66. All constructs were used to transform the recipient strain EGY48 with p8op-lacZ. Transformants were selected on *Ura*/*His* depleted plates at 30°C for 3 days. The activation ability was assayed on Gal/Raf (*Ura^−^*/*His^−^*)/X-gal to test the activation of the *LacZ* reporter gene for 3 days.

### Affymetrix GeneChip hybridization and data analysis

In order to generate gene expression profiles of WT and the *pho* mutant, we conducted 57K Affymetrix rice whole genome array. The total RNA of rice panicle (5–8 cm) samples was isolated using TRizol reagent (Invitrogen) and purified using Qiagen RNeasy columns (Qiagen). All the processes for cDNA and cRNA synthesis, cRNA fragmentation, hybridization, staining, and further scanning, were conducted according to the GeneChip Standard Protocol (Eukaryotic Target Preparation, Affymetrix). 5 ug of total RNA was used for making biotin-labeled cRNA targets. 10 ug of cRNA was hybridized for 16 h at 45°C on GeneChip Rice Genome Array. GeneChips were washed and stained in the Affymetrix Fluidics Station 450. GeneChip were scanned using the Affymetrix GeneChip Scanner. The information about GeneChip Rice Genome Array (MAS 5.0) could be accessed from Affymetrix website: http://www.affymetrix.com/products_services/arrays/specific/rice.affx. GCOS software (Affymetrix GeneChip Operating Software) was used for data collection and normalization. The overall intensity of all probe sets of each array was scaled to 500 to guaranty that hybridization intensity of all arrays was equivalent, each probe set was assigned with present “P”, absent “A” and marginal “M” and p-value from algorithm in GCOS. The microarray data has been deposited in the Gene Expression Omnibus (GEO) of NCBI under accession GSE17194.

### Phylogenetic analysis

All MADS-box proteins were retrieved by BLAST searches using the conserved M-, I-, K-domain regions (174 amino acids) of OsMADS15 protein (http://www.ncbi.nlm.nih.gov). Protein sequences were aligned using the CLUSTALX 1.83 [32]. The phylogenetic tree was constructed using the Molecular Evolution and Genetic Analysis (MEGA) package version 3.1 [33].

### Morphological analysis

For SEM, samples were fixed overnight at room temperature with 2.5% glutaraldehyde in a 0.1 M phosphate buffer (pH 7.4) and dehydrated through an ethanol series. Then the samples were replaced by isoamyl acetate, critical point dried, sputter coated with gold, and observed with a scanning electron microscope. For histology, samples were fixed in FAA (5% formaldehyde, 5% glacial acetic acid and 63% ethanol) overnight at 4°C, dehydrated in a graded ethanol series, embedded in Technovit 7100 resin (Hereaus Kulzer) and polymerized at room temperature. Transverse sections were performed using an Ultratome III ultramicrotome (LKB), stained with 0.25% toluidine blue (Chroma Gesellshaft Shaud) and photographed using an Olympus BX61 microscope.

### Quantitative real-time PCR

Total RNA was extracted from rice young panicles (5–8 cm) using TRIZOL reagent (Invitrogen) as described by the supplier. 3 µg RNA was reverse-transcribed with Oligo-dT(18) primer using the superscript II RNaseH reverse transcriptase (Invitrogen). For quantitative real-time RT-PCR, first strand cDNAs were used as templates in real-time PCR reactions using the SYBR Green PCR Master Mix (Applied Biosystems) according to the manufacturer's instructions. The amplification of the target genes were analyzed using the ABI Prism 7000 Sequence Detection System and Software (PE Applied Biosystems). Ubiquitin was used as a control to normalize all data.

### Bisulfite sequencing

Five micrograms genomic DNA isolated from panicles (5–8 cm) was digested with *EcoR*I and *Pst*I. After centrifugation, pellets were dissolved in 50 µL of water, heated at 95°C for 15 min, and quenched on ice. Fifty microliters of NaOH (3 M) was added and incubated at 37°C for 30 min, followed by the addition of 565 µL bisulfite solution to the denatured DNA. Samples were treated at 55°C for 20 h. After being purified using a Wizard DNA clean-up system (Promega), 50 µL bisulfite-treated DNA was added with 5 µL NaOH (3 M) and incubated at 37°C for 15 min. The Bisulfite-treated DNA was precipitated with ammonium acetate and ethanol, and the pellets were dissolved in 50 µL of water. PCR analysis was performed at 50°C using four primer sets (BSP1-4). PCR products were cloned into PMD18-T vectors. Ten clones of each product were sequenced to determine the methylation ratio. Cytosine methylation was only found in the BSP1 region.

## Supporting Information

Figure S1 The plantlets formed in *pho* panicle show normal vegetative growth when explanted in paddy fields. (A) Young plantlets formed in *pho* panicle. (B) The emergence of normal roots in those plantlets after being replanted in field for three days. (C) The spikelet of WT. (D) The spikelets of ACT::RNAi*MADS15* plants.
(1.41 MB TIF)

Figure S2 OsMADS15-GFP fusion protein and Osmads15 (*dep*)-GFP fusion protein in rice protoplast. Bars: 5 µm.
(0.24 MB TIF)

Figure S3 *dep/nsr* double mutant shows a similar pseudovivipary phenotype to that of the *pho* mutant. (A) The panicles of *dep* (left), *nsr* (center) and *dep/nsr* (right) plants. (B) Young plantlet formed in dep/nsr panicle. (C) The emergence of normal roots in this plantlet after being replanted in field for two days.
(1.48 MB TIF)

Figure S4 Phylogenetic tree of deduced amino acid sequences shows that *OsMADS15* is an *AP1/FUL*-like gene. Phylogenetic tree construction was performed using the M, I, and K domains of these proteins.
(0.23 MB TIF)

Figure S5 Alignment of full-length sequences of *OsMADS15* with *AP1/FUL*-like proteins in other grass species and *Arabidopsis*. Black boxes indicate identical amino acids, and gray boxes indicate similar amino acids. The red box indicates the position of the amino acid substitution in *dep* and *pho* mutant.
(3.13 MB TIF)

Figure S6 Palea is more severely affected than lemma in *dep* spikelet. (A–C), SEM of the lemma (A), palea (B) and glume (C) epidermis of WT spikelet; (D–F), SEM of the lemma (D), palea (E) and glume (F) epidermis of severely affected *dep* spikelet. Scale bar is 10 µm in all panels.
(1.75 MB TIF)

Figure S7 New shoots occasionally emerge from dep spikelets. (A) *dep* plant with emerged shoots (white arrows) in some spikelets. (B) *dep* spikelet with an emerging tiller (white arrow) between palea and upper empty glume. (C) SEM of the emerging tiller (white arrow) in *dep* spikelet. The upper empty glume has been removed. Bar is 0.5 mm (D) Tillers formed in *dep* spikelets. (E) The emergence of normal roots in those tillers after replanting in field for two days.
(2.45 MB TIF)

Table S1 The primers used in this study.
(0.04 MB DOC)
