# Supplementary figures and images for: DEP and AFO Regulate Reproductive Habit in Rice
Source: PLoS Genet. 2010 Jan 22;6(1):e1000818. doi: 10.1371/journal.pgen.1000818 (PMC2809758; doi:10.1371/journal.pgen.1000818)

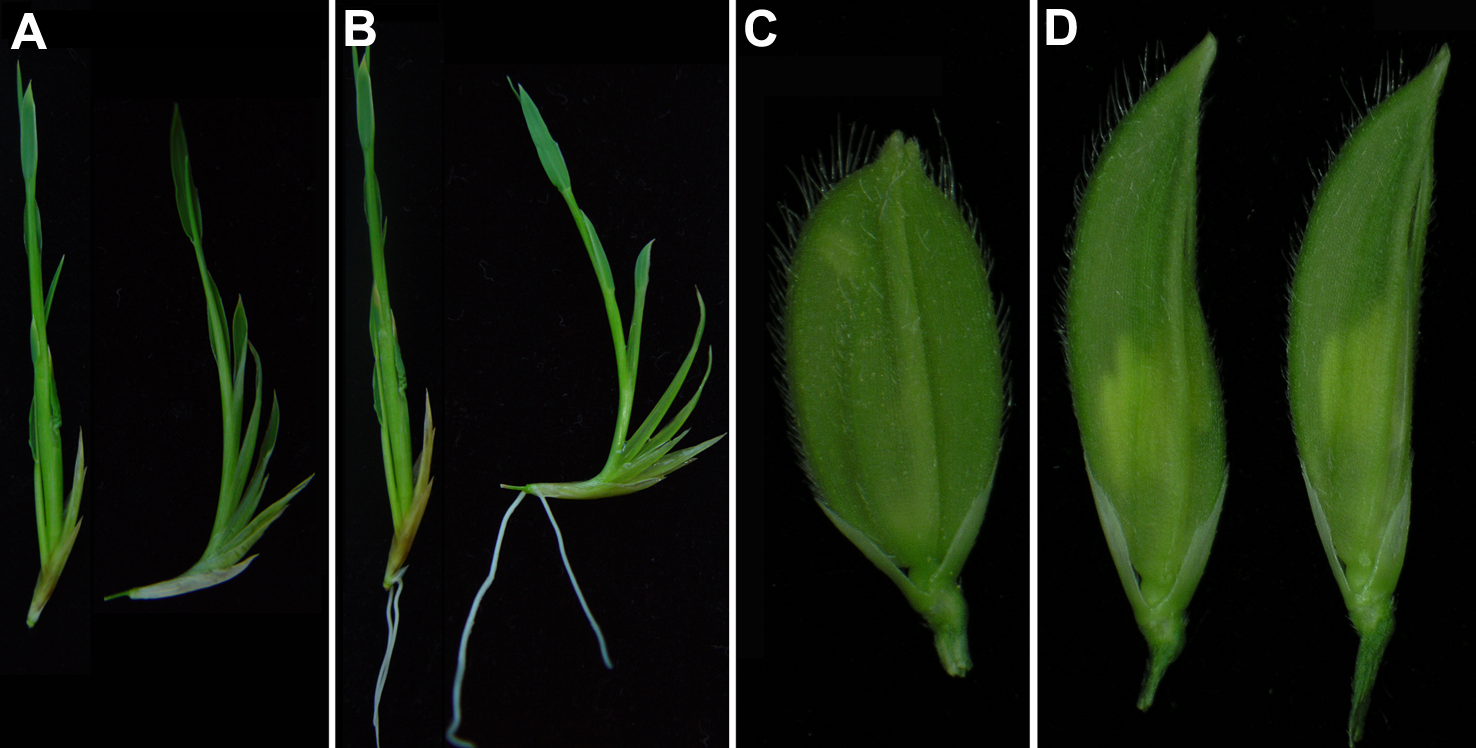

Supplement: Figure S1 — The plantlets formed in pho panicle show normal vegetative growth when explanted in paddy fields. (A) Young plantlets formed in pho panicle. (B) The emergence of normal roots in those plantlets after being replanted in field for three days. (C) The spikelet of WT. (D) The spikelets of ACT::RNAiMADS15 plants. (1.41 MB TIF) [file pgen.1000818.s001.tif]

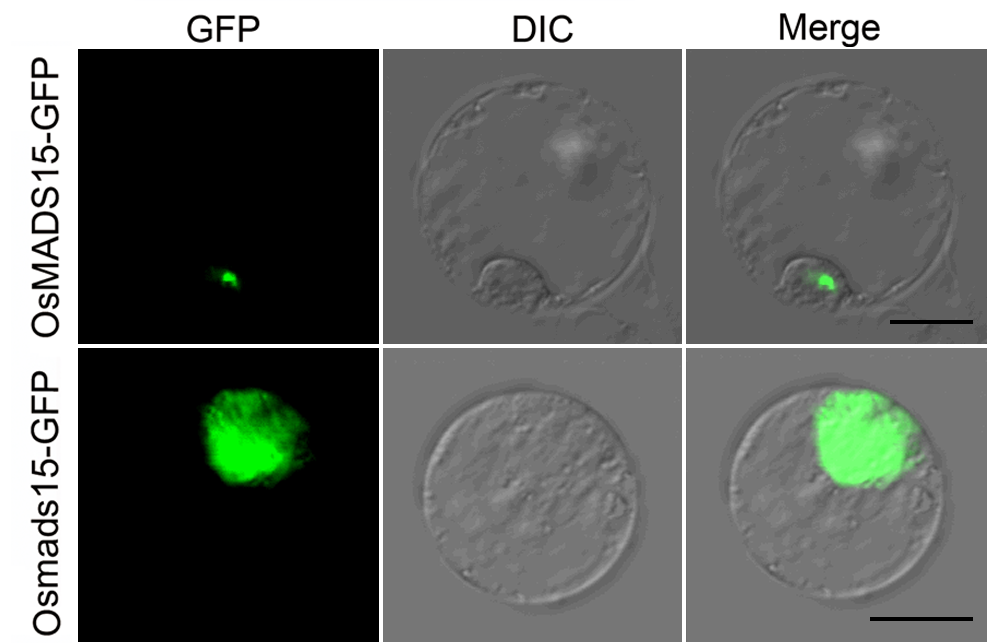

Supplement: Figure S2 — OsMADS15-GFP fusion protein and Osmads15 (dep)-GFP fusion protein in rice protoplast. Bars: 5 µm. (0.24 MB TIF) [file pgen.1000818.s002.tif]

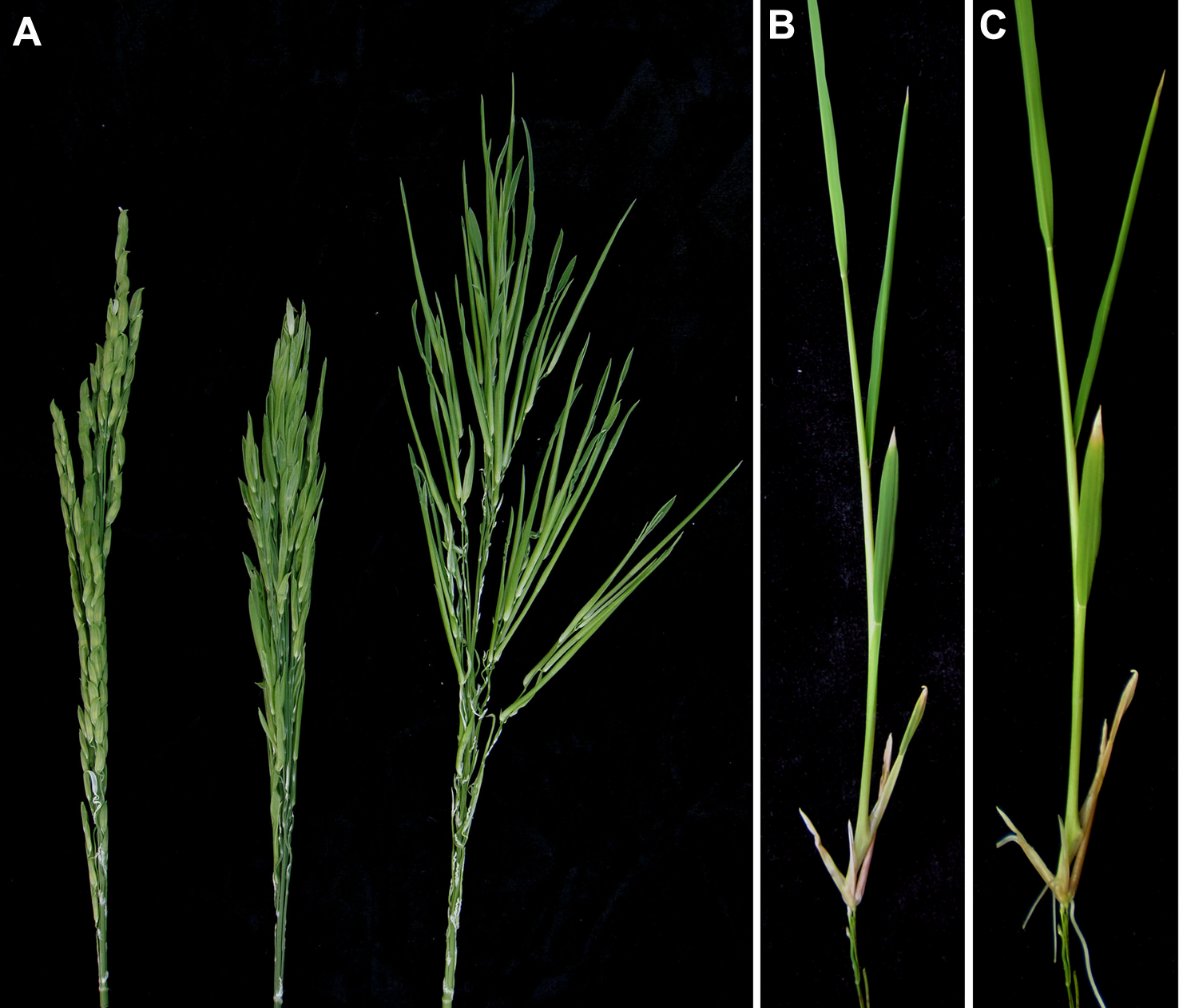

Supplement: Figure S3 — dep/nsr double mutant shows a similar pseudovivipary phenotype to that of the pho mutant. (A) The panicles of dep (left), nsr (center) and dep/nsr (right) plants. (B) Young plantlet formed in dep/nsr panicle. (C) The emergence of normal roots in this plantlet after being replanted in field for two days. (1.48 MB TIF) [file pgen.1000818.s003.tif]

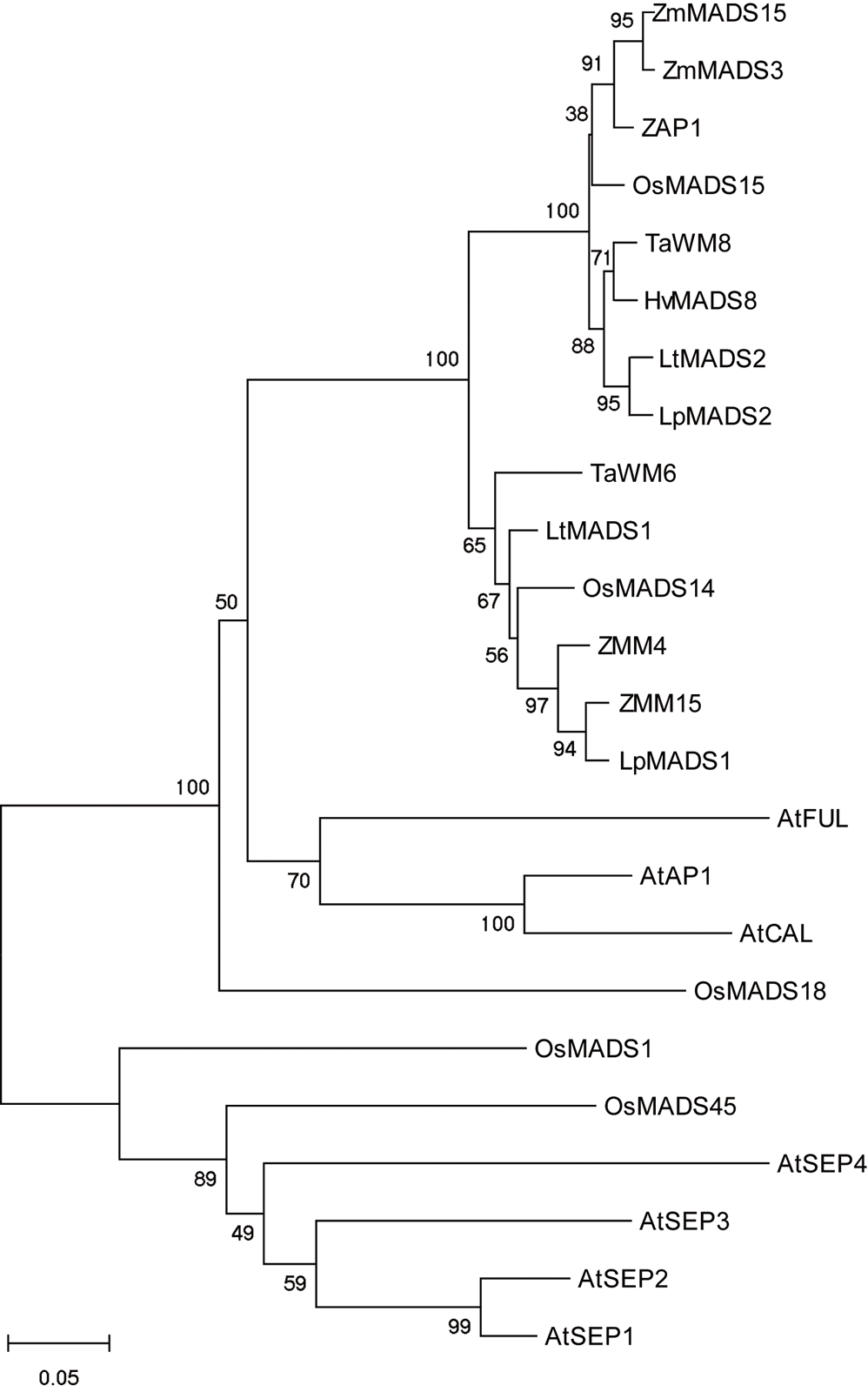

Supplement: Figure S4 — Phylogenetic tree of deduced amino acid sequences shows that OsMADS15 is an AP1/FUL-like gene. Phylogenetic tree construction was performed using the M, I, and K domains of these proteins. (0.23 MB TIF) [file pgen.1000818.s004.tif]

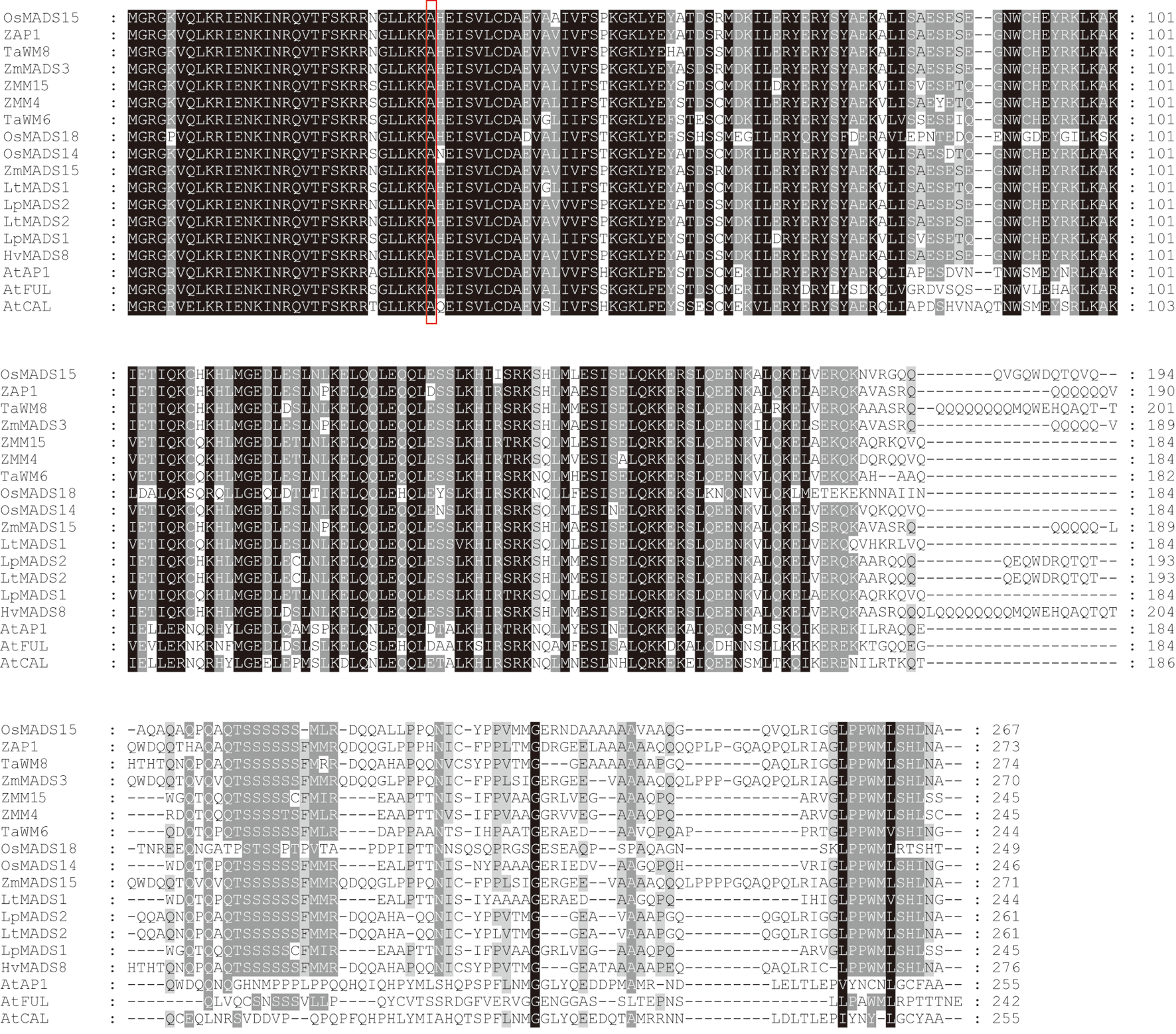

Supplement: Figure S5 — Alignment of full-length sequences of OsMADS15 with AP1/FUL-like proteins in other grass species and Arabidopsis. Black boxes indicate identical amino acids, and gray boxes indicate similar amino acids. The red box indicates the position of the amino acid substitution in dep and pho mutant. (3.13 MB TIF) [file pgen.1000818.s005.tif]

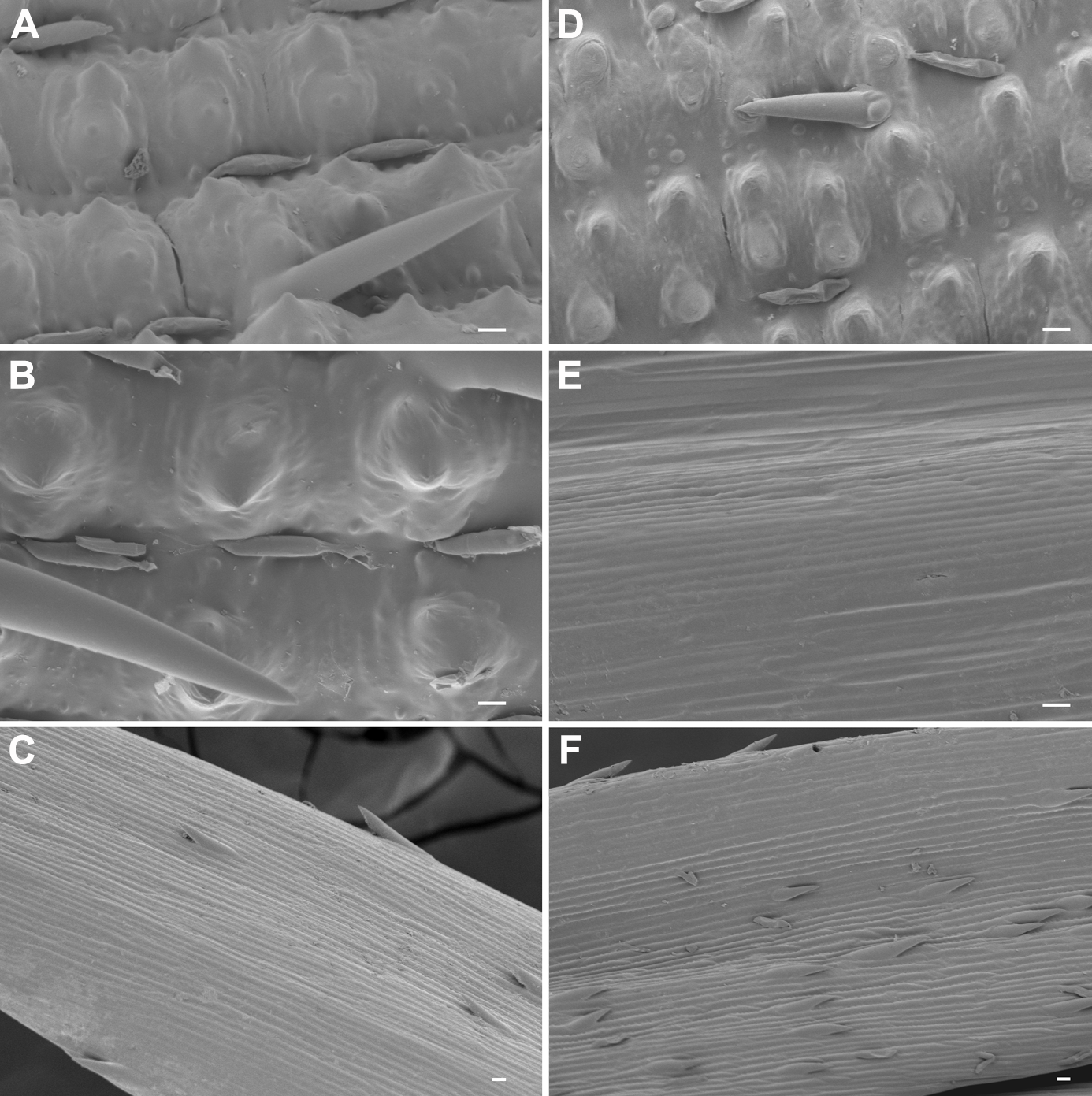

Supplement: Figure S6 — Palea is more severely affected than lemma in dep spikelet. (A–C), SEM of the lemma (A), palea (B) and glume (C) epidermis of WT spikelet; (D–F), SEM of the lemma (D), palea (E) and glume (F) epidermis of severely affected dep spikelet. Scale bar is 10 µm in all panels. (1.75 MB TIF) [file pgen.1000818.s006.tif]

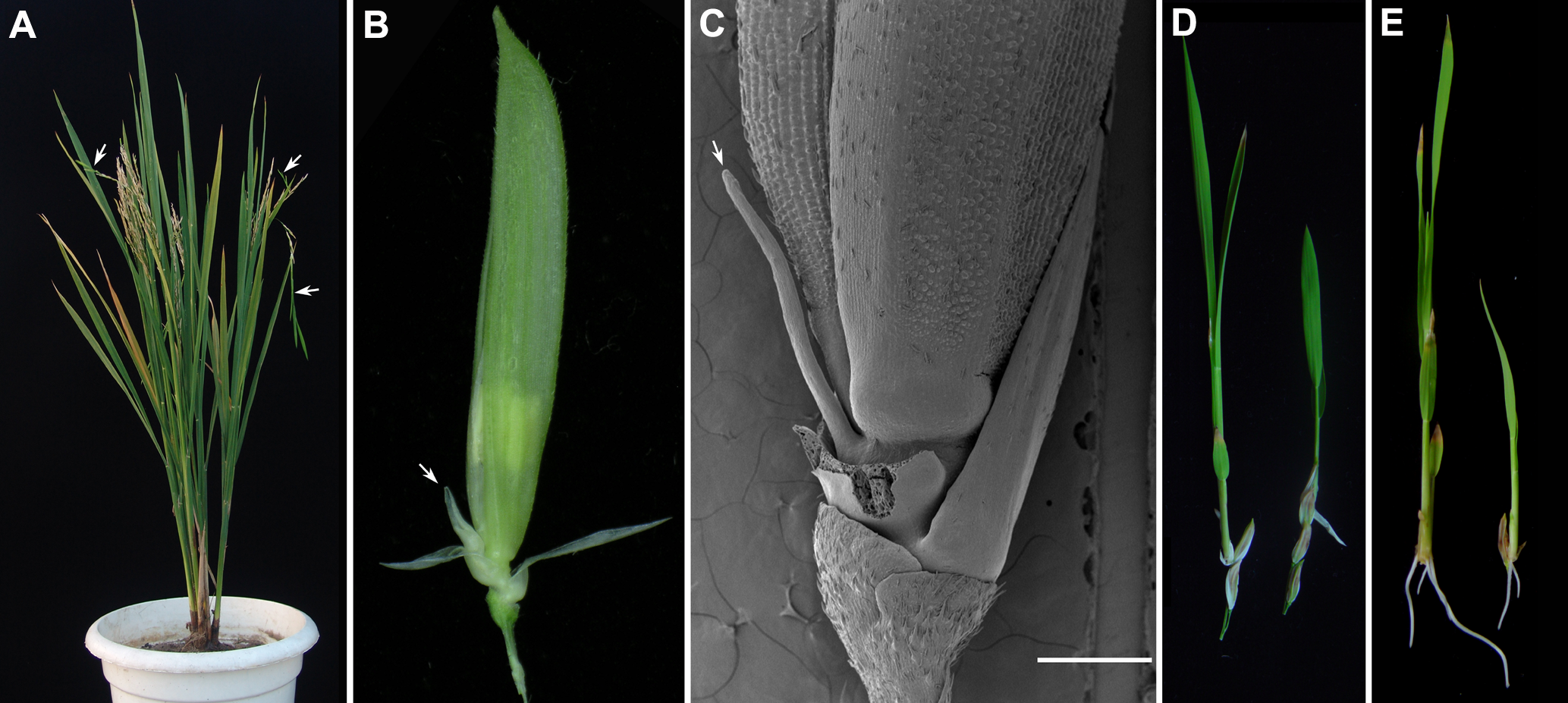

Supplement: Figure S7 — New shoots occasionally emerge from dep spikelets. (A) dep plant with emerged shoots (white arrows) in some spikelets. (B) dep spikelet with an emerging tiller (white arrow) between palea and upper empty glume. (C) SEM of the emerging tiller (white arrow) in dep spikelet. The upper empty glume has been removed. Bar is 0.5 mm (D) Tillers formed in dep spikelets. (E) The emergence of normal roots in those tillers after replanting in field for two days. (2.45 MB TIF) [file pgen.1000818.s007.tif]
