# Supplementary material for: DEP and AFO Regulate Reproductive Habit in Rice
Source: PLoS Genet. 2010 Jan 22;6(1):e1000818. doi: 10.1371/journal.pgen.1000818 (PMC2809758; doi:10.1371/journal.pgen.1000818)
Supplement: Table S1 — The primers used in this study. (0.04 MB DOC) [file pgen.1000818.s008.doc]

| **Name** | **Forward sequence (described 5’ to 3’)** | **Reverse sequence (described 5’ to 3’)** |
| --- | --- | --- |
| P1 | TGGTAGTATGTCCTTAGCAT | CAAGATCGTCCACGTAAGTA |
| P2 | ATTCAATTCCCAAAGAGCAG | GCTGCAACAATCTGTAACTC |
| P3 | ATCACACTCACATCACAGAG | GCAGTTTGACTTCTTTGACC |
| P4 | GTCATCAATCAATTATATCG | GCAGGCTACTTACTAGCGTA |
| P5 | AAGATGAGGTGAGGAAGTGG | AATCACCTACACTATACCGC |
| P6 | ACGATGCGAAAATCCACTTT | ATCAACCATGAGTACACGTCG |
| P7 | GAATCAGCTGTACAAGTTTG | AATTTTGCAGTAGTGGACAT |
| P8 | CTACAGAAGTGCGAGGAACA | GCGATATAGCTCAACCTGAA |
| DEPRNAi | TTGGATCCGCCATGGATGCTGAGCC | TTGTCGACTAGGTACGTGCTGATGATT |
| DEPGFP | TAGAAGCTTAGGTTAGGTCGGA | AGGATCCAGCATTGAGGTGGCTCAGCA |
| DEP-BD | AGAATTCATGGGGCGGGGGAAGGTG | TGTCGACATGATTCACGGTGCATACG |
| DEP-BD2 | CGAATTCCTTGAACGTTATGAGC | AGTCGACATTCTTCTGCCTCTCC |
| LHS1RT | ACCATCAGGGTCTTCTCCAC | GGATGGGATGTGTTCATTGG |
| UBIQRT | CAAGATGATCTGCCGCAAATGC | TTTAACCAGTCCATGAACCCG |
| BSP1 | TTTTAATTAATATAATTTTAATGTATGAAGG | CTCTACCATACCAAACCAAAATATC |
| BSP2 | ATGGAGAGTGGGATTAAATGTTTAA | AAAAACTTCCCTTTCTTCTTCCTAC |
| BSP3 | TGTTAAATTGATAAGGTAGGAAGAAGAA | CTACAACTAAACTAACTAACTTACTCCTAT |
| BSP4 | ATTATAAAGTTAGTTTGTAAAGGGGATAGA | AATTAATTAATTATCTTATTAAACTTAAATC |
